# Human pupillary light reflex during successive irradiation with 1-ms blue- and green-pulsed light

**DOI:** 10.1186/s40101-017-0153-7

**Published:** 2017-10-17

**Authors:** Soomin Lee, Naoko Muto, Yoshihiro Shimomura, Tetsuo Katsuura

**Affiliations:** 1 Center for Environment, Health and Field Sciences, Chiba University, 6-2-1, Kashiwanoha, Kashiwa, 277-0882 Japan; 2 Faculty of Engineering, Chiba University, 1-33, Yayoi-cho, Inage-ku, Chiba, 263-8522 Japan; 3 Graduate School of Engineering, Chiba University, 1-33, Yayoi-cho, Inage-ku, Chiba, 263-8522 Japan

## Abstract

**Background:**

In the human retina, the contribution of intrinsically photosensitive retinal ganglion cells (ipRGCs) to the regulation of the pupillary response remains poorly understood. The objective of the current study was to determine the response dynamics of the pupillary light reflex to short, successive pulses of light. In order to better assess the roles of ipRGCs and cones, we used pulses of blue and green light.

**Methods:**

Each participant was exposed to 1-ms blue (466 nm) and/or green (527 nm) light pulses simultaneously or separately, with inter-stimulus intervals (ISIs) of 0, 250, 500, 750, or 1000 ms. Pupil diameter was measured using an infrared camera system.

**Results:**

We found that human pupillary light responses during simultaneous irradiation or successive irradiation with ISIs ≤ 250 ms were equivalent, though successive irradiation of blue- and green-pulsed light with ISIs ≥ 500 ms induced markedly increased pupillary constriction.

**Conclusions:**

We propose that this result may be related to cell hyperpolarization that occurs in the retina just after the first light stimulus is turned off, with the threshold for this effect being between 250 and 500 ms in the human retina.

## Introduction

In the early part of this century, a novel type of photocell, the intrinsically photosensitive retinal ganglion cell (ipRGC), was discovered in the mammalian retina [1]. Subsequently, ipRGCs were shown to play important roles in non-image-forming responses, including the suppression of pineal melatonin synthesis [2–5], pupillary constriction [4, 6–16], and alertness [17–19]. Research has also demonstrated that ipRGCs respond to short wavelength light of around 480 nm [1, 20, 21].

Recently, researchers have also suggested that input from cones and rods influences ipRGC response [4, 6, 10, 11, 21, 22]. Thus, ipRGCs as well as cones, rods, bipolar cells, amacrine cells, and ganglion cells in the retina are responsible for the pupillary light reflex [23]. Indeed, output from ipRGCs regulates the pupillary light reflex via the olivary pretectal nucleus in the pretectum [21]. Gamlin et al. [6] measured pupillary light reflexes and intracellular electrical impulses in macaque (*Macaca mulatta*) ipRGCs in response to a 10-s pulse of light (493 nm, 13.3 log photons/[cm^2^ s]) and revealed that ipRGCs depolarized rapidly (latency to first spike, 35 ms) and exhibited sustained firing for the duration of the light stimulus. After the light was turned off, brief, transient hyperpolarization was observed, canceling the sustained intrinsic response. The cells then depolarized in darkness to give rise to a sustained late discharge before slowly returning to their resting potential [6]. Given the rapid response of the macaque ganglion cells to light, it is of interest to determine the dynamics of the human pupillary response to short-pulsed light, where the light stimulus on/off phases occur almost simultaneously.

Currently, the temporal summation properties of the pupillary response to successive two-pulse stimulation remain unknown. A prior study [24] on the pupillary response to successive two-pulse stimulation found that the pupillary response increased in amplitude when the inter-stimulus interval (ISI) was less than ~ 600 ms. More recently, Joyce et al. [25] examined whether the post-illumination pupillary response (PIPR) and pupillary constriction were dependent upon the ISI (0, 64, 256, 512, or 1024 ms) between two successive pulses (16 and 100 ms) of blue (464 nm) or red (638 nm) light stimuli with two irradiances (11.4 and 15.2 log photons/[cm^2^ s]). They found that the 6-s PIPR (pupil size at 6 s after light offset) was independent of the ISI and that the maximum pupillary constriction increased as the ISI increased [25].

On the other hand, the effects of simultaneous light exposure on the pupillary response appear to be different from those of successive two-pulse stimulation. For example, Figueiro et al. [22] revealed that compared to monochromatic light exposure to blue (450 nm) or green (525 nm) light, simultaneous exposure to blue and green light resulted in less melatonin suppression. Moreover, we also found that pupillary constriction in response to simultaneous exposure to an extremely short pulse (1 ms) of blue and green light was less pronounced than during exposure to a pulse of blue light alone despite the double irradiance intensity of the combination [26]. These results seem to support the theory that input from rods and cones influences the responses of ipRGCs.

It should be noted, however, that the previous studies on the pupillary response to successive two-pulse stimulation [24, 25] did not successively present pulses of different wavelengths, which might differentially affect ipRGCs, cones, and rods. Therefore, the present study investigated the effects of both simultaneous and successive exposure to blue and/or green light with regard to non-image-forming responses, such as the pupillary light reflex, using extremely short pulses (1 ms) of blue and green light with ISIs ranging from 0 to 1000 ms.

## Methods

### Participants

Nine healthy young Japanese women (mean age 22 ± 0.3 years) participated in the experiment. All participants were confirmed to have normal color vision using the Farnsworth Munsell 100 Hue Test. Written informed consent was obtained from each participant after the procedures of the experiments had been explained to them. This study was approved by the Bioethics Committee of the Graduate School of Engineering at Chiba University (no. 26-33). This research was performed in accordance with the Code of Ethics of the World Medical Association (Declaration of Helsinki) for studies involving humans.

### Experimental environment and apparatus

**Fig. 1:**
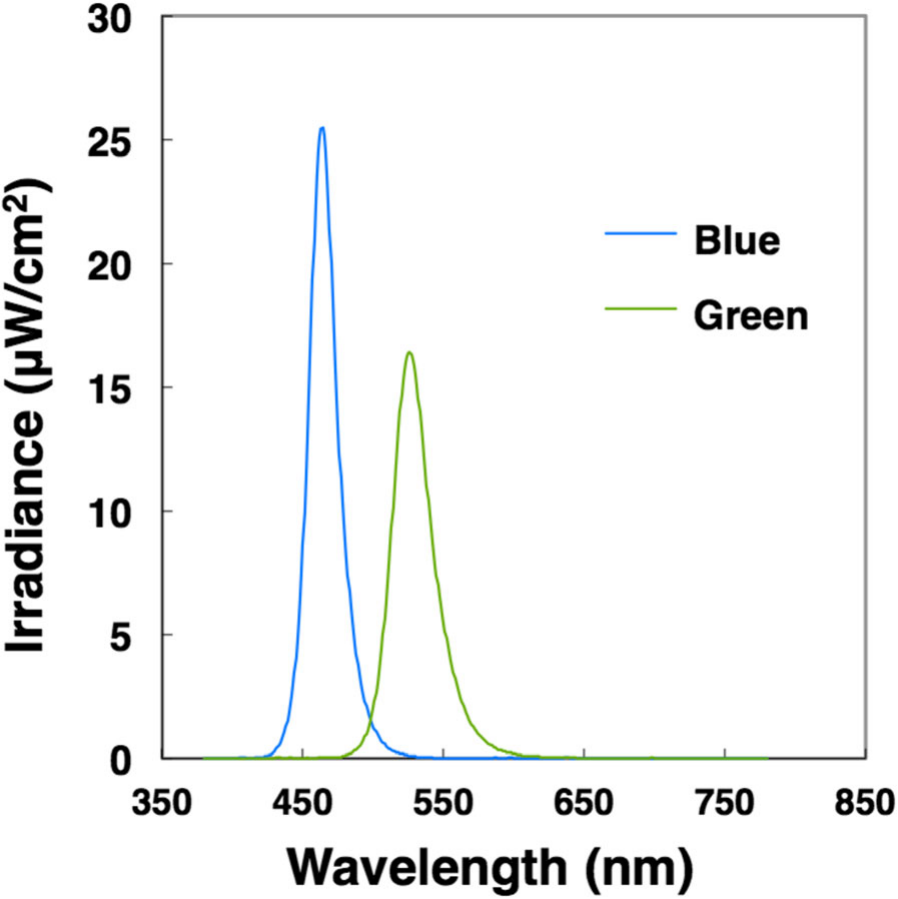
Spectral irradiance with blue and green light

**Fig. 2:**
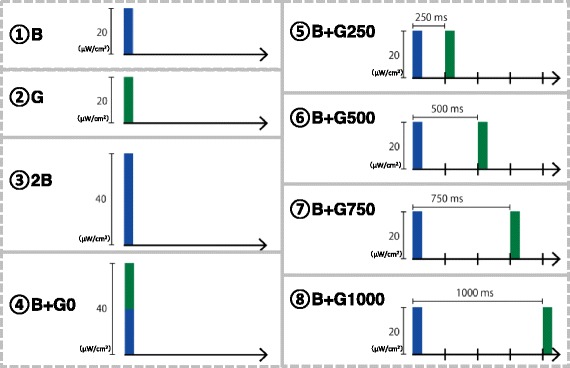
Eight light conditions. Blue bar: pulse of blue light, Green bar: pulse of green light

The experiments were conducted in a climatic chamber (TBR-6HA4G2C; ESPEC Corp., Japan) with an air temperature and relative humidity of 25 °C and 50%, respectively. During the experiment, participants were exposed to extremely short pulses of blue and green light simultaneously or separately using an integrating sphere (Takano Co., Ltd.) in a booth (1420 mm × 510 mm × 1750 mm) (see Section 2.3 for details on the procedures). The pulse width of the light was 1 ms, and the irradiation intensity was ~ 20 μW/cm^2^ or 13.7 log photons/[cm^2^ s]. The spectral irradiance of blue and green light-emitting diodes was measured at the participant’s eye level using a spectroradiometer (CL-500A; Konica Minolta, Inc., Japan). The wavelength of peak emission was 466 nm (full width at half maximum [FWHM] 22 nm) for blue light and 527 nm (FWHM 29 nm) for green light (Fig. 1). We also estimated the melanopsin-stimulating irradiance and photon density of each light condition at the participant’s retinal level [27] based on the spectral absorption of the crystalline lens [28] and a template [29] that indicated the spectral absorption characteristics of the photopigment with a peak wavelength of 484 nm [1] (Table 1). We generated the rectangular pulsed light using a function generator (WF1946A; NF Corporation, Japan) that was attached to a microcomputer (Arduino UNO; Arduino, USA) and control unit (Takano Co., Ltd., Japan). We monitored the waveform of the pulsed light using a photodiode (S3399; Hamamatsu Photonics, Japan) and photosensor amplifier (C8366; Hamamatsu Photonics). The ISIs of the successive pulses of blue and green light were 0, 250, 500, 750, and 1000 ms. We applied the following eight light conditions: blue light only (B), green light only (G), double intensity blue light (2B), simultaneous irradiation with blue and green light with an ISI of 0 ms (B + G0), and successive irradiation with blue and green light with an ISI of 250 ms (B + G250), 500 ms (B + G500), 750 ms (B + G750), and 1000 ms (B + G1000) (Fig. 2).

**Table 1:** Characteristics of light sources

Light source	Blue	Green
Peak wavelength (nm)	466	527
FWHM (nm)	22	29
Photopic illuminance (lx)	15	105
Scotopic illuminance (lx)	226	241
Irradiance (μW/cm ^2^ )	20	19.1
Photon density (10 ^12^ photons/[cm ^2^ s])	47.3	51.2
Photon density (log photons/[cm ^2^ s])	13.7	13.7
Melanopsin-stimulating photon density (10 ^12^ photons/[cm ^2^ s])	39.6	25.7

*FWHM* full width at half maximum

### Procedure and measurements

**Fig. 3:**
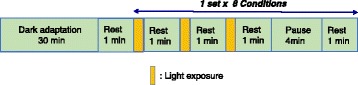
Procedure of the experiment

Each participant sat on a chair with her eyes facing the integrating sphere, which contained the light-emitting diode arrays. After 30 min of dark adaptation (< 0.5 lx), each participant was successively exposed to the eight light conditions with 5-min intervals including 1 min of rest in the dark (Fig. 3). The order of the eight light conditions was counterbalanced across the participants. The pupil diameter (PD) of the left eye was measured for 12 s, starting from 1 s before the onset of the light pulse, using an infrared camera system (EMR-8B; nac Image Technology Inc.). Each participant was exposed to each light condition three times every 60 s. We used the mean value of three time points during each light exposure.

Using the PD measurements, we calculated the percentage (%) of peak pupillary constriction as follows: % peak pupillary constriction = [(baseline PD − minimum PD after light exposure)/baseline PD] × 100 where the baseline PD represents the average value 1 s before light exposure. Additionally, we determined the recovery time, which was defined as the time until 90% recovery of the PD from baseline. We also obtained the peak latency and 6-s PIPR amplitude (% pupil size at 6 s after light offset) from the PD recordings [30, 31].

### Statistical analysis

Using SPSS 23.0 (IBM, USA), we performed one-way repeated measure analyses of variance to evaluate the effects of the light conditions. When any significant effect was found, multiple comparisons between the light conditions were performed according to the Bonferroni procedure. Differences were considered statistically significant at *p* < 0.05.

## Results

**Fig. 4:**
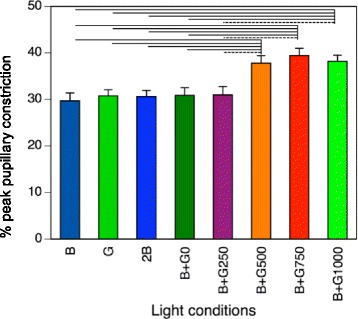
Results for % peak pupillary constriction. Error bars indicate one standard error of the mean. Solid lines denote *p* < 0.01, and broken lines indicate *p* < 0.05

The main effect of light condition was statistically significant (*p* < 0.001, *F* value = 27.425, degree of freedom *=* 7) in the % peak pupillary constriction. The % peak pupillary constriction after exposure to extremely short monochromatic light (B, G, 2B) and simultaneous pulses of blue and green light (B + G0) was approximately 30% (Fig. 4). The actual peak pupillary constriction was ~ 1.7 mm, with peak latencies observed around 1 s after irradiation in these conditions (Table 2).

**Table 2:** Peak pupillary constriction, peak latency, 6-s PIPR amplitude, and 90% recovery time (mean ± SD)

	Peak pupillary constriction (mm)	Peak latency (s)	6-s PIPR amplitude (% baseline)	90% recovery time (s)
B	1.71 ± 0.03 ^a^	1.02 ± 0.08 ^c^	95.8 ± 1.64	2.83 ± 0.72
G	1.74 ± 0.18 ^b^	1.00 ± 0.10 ^c^	95.4 ± 1.38	2.82 ± 0.36 ^f,h^
2B	1.73 ± 0.23 ^c^	1.04 ± 0.09 ^c^	96.3 ± 2.21	2.90 ± 0.60 ^h^
B + G0	1.73 ± 0.24 ^c^	1.02 ± 0.07 ^c^	96.2 ± 2.71	2.70 ± 0.59 ^g,h^
B + G250	1.75 ± 0.32 ^d^	1.05 ± 0.21 ^e^	96.2 ± 2.19	2.80 ± 0.87 ^h^
B + G500	2.14 ± 0.31	1.29 ± 0.10 ^e^	94.3 ± 4.11	3.57 ± 0.56
B + G750	2.24 ± 0.03	1.52 ± 0.06	94.8 ± 2.62	4.03 ± 0.85
B + G1000	2.16 ± 0.25	1.77 ± 0.08	95.6 ± 2.20	4.22 ± 0.83
ANOVA *(p* value, *F* value*)*	*p <* 0.001, *F =* 21.976	*p* < 0.001, *F* = 111.745	*p* = 0.418, *F* = 1.034	*p* < 0.001, *F* = 10.450

*ANOVA* effect of the light conditions in each measurements

a~h: the results of mutiple comparison

^a^significantly smaller than B+G500 (*p* < 0.01), B+G750 (*p* < 0.05) and B+G1000 (*p* < 0.05)

^b^significantly smaller than B+G500 (*p* < 0.05), B+G750 (*p* < 0.05) and B+G1000 (*p* < 0.01)

^c^significantly smaller (*p* < 0.01) than B+G500, B+G750 and B+G1000

^d^significantly smaller (*p* < 0.05) than B+G500, B+G750 and B+G1000

^e^significantly smaller (*p* < 0.01) than B+G750 and B+G1000

^f^significantly smaller (*p* < 0.01) than B+G500

^g^significantly smaller (*p* < 0.05) than B+G750

^h^significantly smaller (*p* < 0.01) than B+G1000

**Fig. 5:**
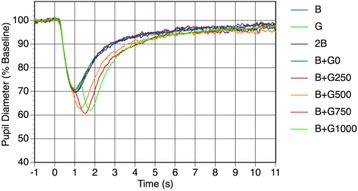
Waveforms of pupillary diameter during each light condition

The % peak pupillary constriction following exposure to successive irradiation with pulses of blue and green light with ISIs of 500, 750, and 1000 ms (B + G500, B + G750, and B + G1000) was pronounced (Fig. 4). Specifically, the % peak pupillary constriction during the B + G500, B + G750, and B + G1000 conditions reached more than 37%. The actual mean pupillary constriction of these conditions was > 2.0 mm, and the peak latencies of the B + G500, B + G750, and B + G1000 conditions occurred at 1.29, 1.52, and 1.77 s, respectively, after irradiation, which were significantly larger than those of B, G, 2B, and B + G0 (Table 2). The waveforms of pupillary diameter during the successive irradiation conditions of B + G750 and B + G1000 were bimodal (Fig. 5). In contrast, the pupillary constriction in the B + G250 and B + G500 conditions exhibited a single waveform, even though the pulsed light was presented twice.

Our analyses showed that successive irradiation with pulses of blue and green light at ISIs ≥ 500 ms induced significantly greater pupillary constriction than did the other conditions (*p <* 0.05 or *p* < 0.01; Fig. 4). However, conditions with ISIs ≤ 250 ms exhibited the same level of % peak pupillary constriction as the B + G0 condition. The recovery times for the B, G, 2B, B + G0, and B + G250 conditions were all around 2.8 s, which was shorter than was any of the recovery times for the light conditions with ISIs ≥ 500 ms (Table 2). However, the 6-s PIPR amplitudes of all eight light conditions were around 95%, and no significant differences were identified between the conditions (Table 2).

## Discussion

In the present study, we observed that the pupillary light reflex elicited by a 1-ms pulse of light was prolonged (e.g., the recovery time in response to B was 2.9 s). This prolonged response may be related to the nature of ipRGCs. Researchers have suggested that pupillary constriction is mainly controlled by rods under exposure to light of lower irradiance light and by ipRGCs under exposure to light of higher irradiance [4, 11, 13, 27]. McDougal and Gamlin [32] reported that the contribution of rods, cones, and ipRGCs to the pupillary constriction was affected by the duration of a light stimulus. Specifically, they found that the human pupillary constriction that occurs in response to lower irradiance light (~ 11.0 log photons/[cm^2^ s]) of 10 s or shorter depended mainly on the rod photoresponse, while the pupillary response to such weak stimuli of 30 s or longer depended on the melanopsin photoresponse of ipRGCs [32]. The authors also found that ipRGCs contributed more than did rods or cones to the pupillary constriction when participants were exposed to higher irradiance light (~ 12.5 log photons/[cm^2^ s]) that was < 2 s in duration [32]. Therefore, we assumed that the pupillary constriction in the present study was mainly activated by ipRGCs.

Our data also revealed that the pupillary constriction that occurred in response to B, G, 2B, and simultaneous pulses of blue and green light (B + G0) was almost identical. In a previous study involving macaque monkeys, pupillary constriction in response to 532-nm light exposure was saturated by light with an irradiance intensity of > 13.7 log photons/[cm^2^ s] [6]. The same study also examined the pupillary response to light at 10 wavelengths between 430 and 613 nm and found that the pupillary constriction data obtained for stimuli between 452 and 552 nm were comparable [6]. Hence, the present results might be expected given the irradiance intensity and wavelength of the light we used and may further suggest that human pupillary constriction may be affected by cones as well as ipRGCs.

In the current study, we observed pupillary constriction in response to the first pulse of blue light for 2.9 s, with the peak occurring approximately 1 s after irradiation. Thus, in the conditions that utilized successive irradiation, the second stimulus to reach the retina may have evoked a weaker response that was in proportion to the decreased pupil area. For example, if the pupil area is indeed the dominant determinant of the pupillary reflex, the pupillary constriction that occurred following the second irradiation in the B + G1000 condition should have been inhibited. However, we found that the pupillary constriction to the second irradiation in the B + G1000 condition was actually the most prominent, whereas no pupillary constriction was noted in response to the second irradiation in the B + G250 condition. These findings imply that the degree of pupillary constriction that occurs during successive irradiation is not accounted for by the pupil area and may be affected instead by electrical activity in the retinal cells.

Although the pupillary constriction we observed in response to the successive irradiance of the B + G250 condition was not different from the pupillary constriction we observed in response to the simultaneous irradiation of the B + G0 condition, the pupillary constriction we identified during the conditions with ISIs ≥ 500 ms was remarkable. In a previous study on pupillary constriction in response to two successive light pulses (10-ms duration), the pupillary constriction was slightly increased when the ISI was < 250 ms and continued to increase as the ISI increased to 600 ms [24]. Similarly, another study showed that the pupillary constriction amplitude in response to two 100-ms pulses tended to increase as the ISI increased up to 1024 ms under four melanopsin excitations (blue or red with 15.2 or 11.4 log photon/[cm^2^ s]) [25]. Notably, the pupillary constriction the authors observed in response to pulses of lower-intensity blue light increased with increasing ISIs up to 512 ms and then plateaued through an ISI of 1024 ms [25], which is in accordance with the results of the present study. The irradiance intensity of the light we applied in the present study was higher than was that used in Joyce’s study; additionally, the pulse width was much shorter and the wavelength of the second pulse was different. Given these factors, we may assume that the influence of light on ipRGCs was comparable.

Research has demonstrated that the 6-s PIPR amplitude is the most applicable index for studying ipRGC function [31]. Moreover, it is well known that the 6-s PIPR amplitude varies as a function of the properties of stimuli. For instance, stimuli that have a blue component [30, 31, 33], are longer in duration [31, 33], and have a higher intensity [31] induce larger 6-s PIPR amplitudes than do other stimuli. However, in the present study, we did not find any differences in the 6-s PIPR amplitude among the single or simultaneous irradiation conditions (B, G, 2B, and B + G0). Interestingly, the peak pupillary constriction levels were the same among these conditions. Therefore, we speculate that the influence of light was saturated in these conditions. In addition, we found that the 6-s PIPR amplitudes were identical in the successive irradiation conditions with ISIs between 0 and 1000 ms. Previously, Joyce et al. [25] reported that the 6-s PIPR amplitude was independent of the ISI (0, 64, 256, 512, and 1024 ms) for both blue and red stimuli with two irradiance levels, which is consistent with the findings of the present study.

Finally, we identified substantial differences between the responses elicited by the B + G250 condition and those elicited by the B + G500 condition. In a previous study, intracellular electrical recordings from macaque ipRGCs following a 10-s pulse of light revealed that ganglion cells undergo depolarization followed by transient hyperpolarization (likely less than a few hundred milliseconds) just after the light has been turned off [6]. As such, we hypothesize that in our successive irradiation conditions, the second light stimulus, when presented immediately after the first stimulus, did not cause further pupillary constriction owing to the hyperpolarized state of the cell. The results of the present study indicate that the threshold for this effect in the human retina lies between 250 and 500 ms.

## Conclusions

Collectively, our results indicate that the human pupillary light responses to simultaneous and successive irradiation with pulses of blue and green light at ISIs ≤ 250 ms were equivalent. On the other hand, successive irradiation with pulses of blue and green light at ISIs ≥ 500 ms induced pronounced pupillary constriction. We conclude that this result may be related to hyperpolarization of the retinal cells, which likely occurred immediately after the first light stimulus was turned off; further, our data suggest that the threshold for this effect is between 250 and 500 ms. Altogether, we think that our findings improve our understanding of human pupillary light responses.
